# Chronic Sleep Restriction Increases Negative Implicit Attitudes Toward Arab Muslims

**DOI:** 10.1038/s41598-017-04585-w

**Published:** 2017-06-27

**Authors:** Anna Alkozei, William D. S. Killgore, Ryan Smith, Natalie S. Dailey, Sahil Bajaj, Monika Haack

**Affiliations:** 1 Department of Psychiatry, University of Arizona College of Medicine, Tucson, AZ, USA; 2 McLean Hospital, Harvard Medical School, Belmont, MA, USA; 3 Department of Neurology, Beth Israel Deaconess Medical Center, Harvard Medical School, Boston, MA, USA

## Abstract

Chronic sleep restriction is a common experience; and while it has negative physiological effects, little is known about how it affects human behavior. To date, no study has investigated whether chronic sleep restriction can influence implicit attitudes (e.g., towards a race). Here, in a randomized, counterbalanced crossover design, we subjected participants to 3 weeks of chronic sleep restriction in the lab (i.e., 3 weekly cycles of 5 nights of 4 hours of sleep per night followed by 2 nights of 8 hours of sleep) and found evidence for an increased negative implicit bias towards Arab Muslims. No indicators of an implicit bias were found in these same individuals when they were rested (during a counterbalanced 3-week period of 8 hours time in bed per night). These findings suggest that chronic sleep restriction may “unmask” implicit racial or ethnic biases that are otherwise inhibited when in a rested state. Because chronic sleep restriction is prevalent among many occupations that routinely interact with ethnic minorities in potentially high-conflict situations (e.g., police officers), it is critical to consider the role that restricted sleep may play in exacerbating negative implicit attitudes and their potential for provoking unintentional and potentially harmful behavioral consequences.

## Introduction

The impact of negative attitudes or biases towards ethnic and racial minorities on discriminatory behavior in the workplace, criminal investigations, and judicial proceedings, has received considerable attention in recent years^1^. While most individuals explicitly deny significant negative biases towards ethnic minorities, such biases are often apparent when measured implicitly^2^. The Implicit Association Test (IAT)^3^ is a well-validated^4, 5^ and widely used assessment of an individual’s implicit attitudes and beliefs (e.g., toward a group of individuals from a certain ethnic or religious background). The IAT specifically assesses the differential strength of cognitive associations between various concepts (e.g., “African Americans” and “Bad” versus “African Americans” and “Good”) as a measure of implicit bias. Importantly, some studies have found that stronger implicit biases towards specific groups of individuals (e.g., racial minorities, such as African Americans) are associated with greater amounts of explicit hostile/discriminatory behavior as well, even by individuals of that same minority group^6–8^. Recently, in line with the current political and social climate, research has focused on how implicit biases toward Arab Muslims may affect explicit discriminatory behavior. For instance, the probability of deciding to invite someone with an Arab Muslim name versus a Swedish name (with identical resumes) to a job interview decreased by 5 percent when the recruiter had a stronger negative implicit bias towards Arab-Muslim names^9^. While this may sound like a numerically small effect, such subtle biases may still have profound societal impact when projected across a large proportion of the population or when repeated over multiple occasions^10^. In addition, such implicit biases may have especially severe or fatal consequences in some high-conflict situations; for example, studies using computerized game-style tasks have shown that participants are more likely to shoot an armed individual wearing a traditionally Islamic head dress than an otherwise identical individual without such headgear^11, 12^. However, other work suggests that the effect sizes within these studies are small, and the societal significance of these findings is not yet known; therefore, while potentially important, the practical significance of these findings should be assessed with caution^13^.

Nonetheless, it is well-known that decision-making can be influenced by a number of factors that are outside conscious awareness^14^. In particular, growing evidence suggests that decision-making can be affected by insufficient sleep^15, 16^. Acute total sleep deprivation for two continuous days has been found to increase the tendency to blame others for problems^17^, reduce global emotional regulation^18^, and negatively impact the quality of emotionally-based moral decision-making (i.e., deciding the fate of others during dilemmas involving serious harm or death) while keeping other non-emotional executive functions intact^16^.

While total sleep deprivation clearly affects many aspects of emotional judgment and decision-making, it is even more critical to understand how the effects of chronic partial sleep restriction, a much more prevalent pattern of sleep curtailment, may influence these processes. The National Sleep Foundation recommends between 7–9 hours of sleep per night for most healthy adults^19^. However, around 44% of Americans actually get less than 7 hours of sleep on workdays, and tend to “catch up” on sleep during weekend nights^20^. This pattern is even more severe among individuals in shift-work occupations, who commonly report even shorter sleep durations (i.e., less than 6 hours^20, 21^). Given that many of the most sleep restricted shift-work occupations such as police officers^22, 23^ and military personnel^24^ routinely interact with ethnic minority individuals in potentially high stakes or high-conflict situations that require split-second decisions, it is critical to understand whether the prevalent pattern of sleep restriction affects the expression of subtle implicit racial or ethnic biases.

**Figure 1:**
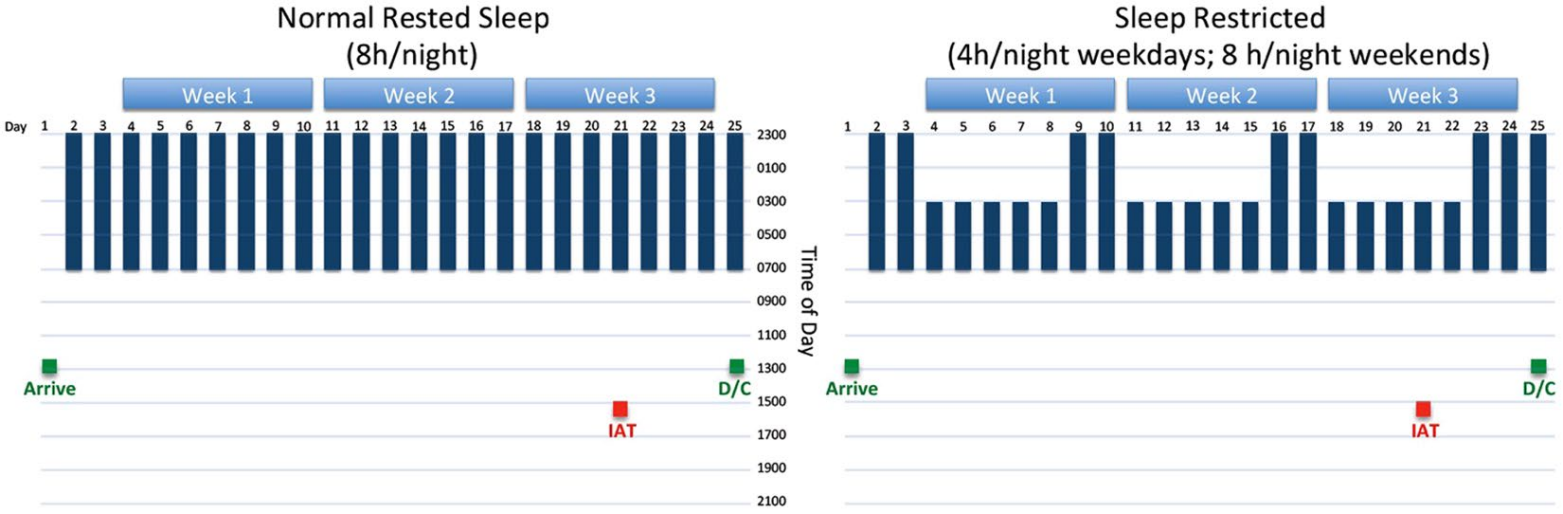
Diagram presenting the sleep and testing schedules for the sleep control and sleep restricted conditions. Sleep Restriction and Normal Rest conditions each comprised 25 days of confinement to a sleep laboratory. IAT = Implicit Association Test; D/C = Discharge from study condition.

To experimentally model the prevalent cyclical pattern of chronic sleep restriction/make-up sleep, we asked participants to undergo a within-subject, counter-balanced sleep restriction study involving two 25-day stays confined to a sleep laboratory. Seventeen healthy men and women were randomly assigned to two alternate conditions that included: 1) 3 weekly cycles comprising 5 days of 4 hours of sleep per night (0300–0700 h) followed by 2 nights of 8 hours of sleep (2300–0700 h), and 2) a matched 3-week control condition comprising 8 hours of sleep opportunity per night (2300–0700 h). In a randomized crossover design, each participant served as his or her own control by completing both 25-day conditions in counterbalanced order (see Fig. 1). We administered the Arab-Muslim Names IAT on two occasions, comprising Day 21 of the sleep-restricted and Day 21 of the sleep control condition. IAT d-scores were calculated for each participant for the two conditions according to the standard algorithm (see Method)^25^. A d-score of zero suggest that no IAT effect is present, whereas a positive IAT score suggest a small (~0.15), moderate (~0.35) or large (~0.65) IAT effect^25^. We hypothesized that after three weeks of chronic sleep restriction, participants would display a significantly stronger IAT effect than after three weeks of control sleep.

## Results

**Figure 2:**
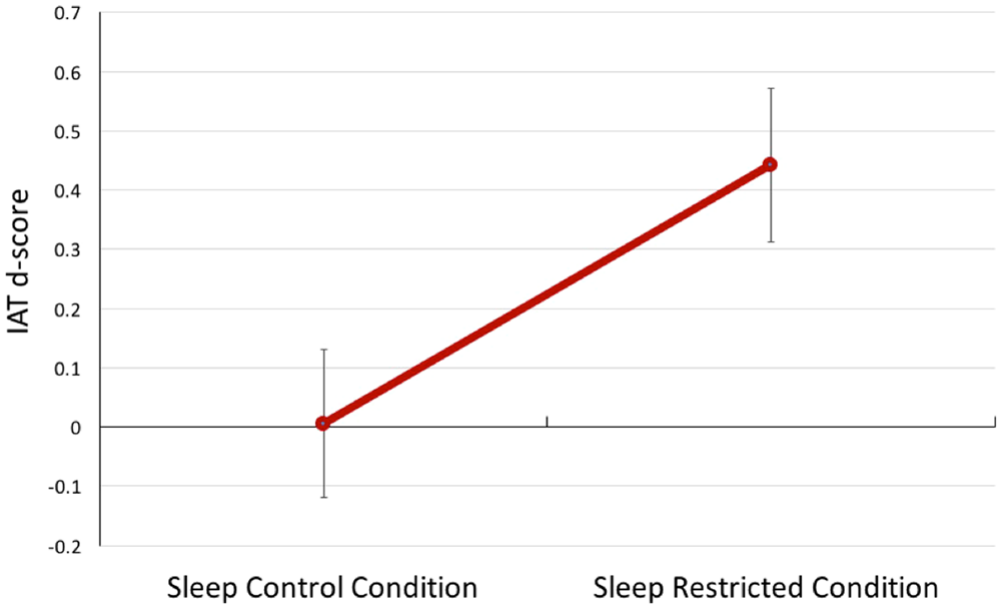
This figure represents the estimated marginal means with standard error (± 1 SE) bars for IAT d-scores for the sleep control (n = 15) and sleep restricted conditions (n = 14).

Change in IAT d-scores between the sleep control and the sleep restricted condition were analyzed using a linear mixed model. Two participants had missing data for the sleep control condition and three participants had missing data for the sleep restricted condition, leaving twelve participants with full datasets from both time points. A two-sided linear mixed-model was chosen to analyze change in d-scores between the sleep control and sleep restricted condition as an alternative to a paired t-test, as the mixed model has the advantage of using all available data and does not employ a listwise deletion. The mixed model showed a significant main effect of sleep condition (F(1, 27) = 5.88, p = 0.022, d = 0.92). While no evidence for an IAT effect was found when participants were rested (d = 0.006, SD = 0.12), participants showed a moderate IAT effect when sleep restricted (d = 0.443, SD = 0.13) (see Fig. 2).

## Discussion

In support of our a priori hypothesis, participants showed an increase in their negative implicit bias towards Arab-Muslim names after being chronically sleep restricted to 4 hours per night for three weeks. Surprisingly, our results did not show an “IAT effect” in our sample when fully rested (i.e., they did not display evidence of implicit negative attitudes towards Arab-Muslim names relative to Other People control names in this condition). These findings imply that even among individuals who do not appear to show a negative implicit bias during the rested condition, this bias emerges clearly when sleep is restricted. Such findings have obvious implications for individuals who operate in high-stakes environments or must make critical decisions about others under conditions of chronically limited sleep.

The fact that an IAT effect emerges after sleep restriction is consistent with well established neuroimaging findings showing that sleep loss is associated with significant metabolic declines in the prefrontal cortex, a region of the brain that is critical for self-monitoring and behavioral control^26^. Moreover, sleep loss is associated with reduced functional connectivity between the prefrontal cortex and emotionally responsive regions of the brain such as the amygdala, leading to impaired inhibitory control over emotional responses when sleep is lacking^27^. Prior research in non-sleep deprived individuals has also shown that greater amygdala activation in response to face stimuli of Black individuals is associated with greater negative implicit race bias^28^. Hence, our findings are consistent with the argument that sleep restriction may degrade prefrontal inhibitory systems leading to greater emotional reactivity and conflict during incongruent versus congruent trials on the IAT, a possibility that could be explored more directly in future studies with neuroimaging.

While the present findings suggest that individuals who are sleep restricted are likely to show increased negative biases toward minorities (in this case Arab Muslims), it also raises the possibility that obtaining adequate sleep may serve as a protective factor against expressing such biases. We found no evidence of implicit biases among individuals when they were given the opportunity to obtain a regular 8-hour sleep schedule. The lack of an IAT effect during the rested condition was unexpected, as previous studies have shown evidence for robust IAT effects^29^. However, to our knowledge, previous IAT studies have not generally accounted for participant sleep quality or quantity, highlighting the intriguing possibility that the typical levels of insufficient sleep pervasive in society may have unknowingly contributed to many of the previous findings. As mentioned above, nearly half of individuals in the U.S. obtain less than the recommended 7 hours of sleep per night, and many routinely get far less^20^. Thus, prior findings in normal individuals may potentially reflect the effects of mild chronic sleep debt relative to what was seen here, as participants were given 8 hours sleep opportunity every night for three weeks—an amount that is atypical in modern society. Future studies should therefore investigate further whether lack of sleep may in fact play a major role in “unmasking” implicit biases that may remain undetected when sufficient sleep is obtained.

While there is mixed evidence on whether implicit attitudes predict explicit discriminatory behavior^9, 13^, our findings may have important behavioral implications for a range of individuals who often experience chronic partial sleep restriction. This may be particularly relevant to explore in greater detail as sleep deprivation has been shown to predict other aspects of behavior and decision-making^16, 30^. Due to occupational demands, many people often find themselves chronically restricted of sleep due to shift-work schedules. For a variety of reasons, many of these same occupations (e.g., police, airline security, first responders, healthcare workers, military personnel) also require frequent interactions with individuals from a variety of cultures, ethnicities, and backgrounds. This raises the concern that greater negative implicit biases towards particular groups, elicited by restricted sleep, may lead to unintentional negative influences on the quality of interactions with these individuals, potentially leading to more hostile exchanges and greater conflict.

Our findings argue in favor of the vital importance of obtaining sufficient sleep, particularly for individuals who work in critical decision-making or high-stakes occupations where implicit biases could lead to severe or life-altering consequences. While sleep modification appears to be one clear method for minimizing implicit biases, there is evidence that such biases can also be affected through focused training. For instance, the implicit *shooter bias* toward ethnic or racial minorities can be overcome with extensive behavioral training^31^, and implicit biases can be reduced in individuals who are consciously concerned about their own potential for discriminatory behavior and who practice the strategies they are taught^32^. It remains unclear whether such training programs would be facilitated by efforts to optimize sleep as well. Further research may explore whether bias remediation training can be combined with sleep extension interventions to enhance effectiveness.

It is important to point out that the current study was limited by a relatively small sample size, although the within-subject cross-over design contributed to the power to detect effects. While the study benefits from a longitudinal and naturalistic study design whereby participants underwent a chronic sleep restriction paradigm that reflects the sleep patterns of a significant proportion of the U.S. population, the results nevertheless need to be replicated with more robustly powered samples. In addition, it should be highlighted that while this study focused on negative implicit biases towards Arab Muslims, based on our proposed mechanism (i.e., whereby sleep deprivation reduces cognitive control over implicit biases), chronic sleep restriction would be expected to potentially increase any IAT effect; our findings will therefore need to be replicated in future studies investigating negative implicit biases towards other cultural, ethnic, and minority populations. Finally, it should be pointed out that it is unclear whether the negative implicit bias observed after chronic sleep restriction was directed towards the ethnicity of the names presented, or whether it reflects a negative implicit attitude towards the assumed religion of individuals with such names, or a combination of the two. Future research will be necessary to disentangle these effects in greater detail.

In summary, our findings clearly show that 3 weeks of chronic sleep restriction unmasks a negative implicit bias towards Arab Muslims in individuals who do not characteristically show this type of bias when normally rested. Because our paradigm was designed to mimic the common cyclic pattern of weekday sleep restriction and weekend recovery sleep that is widespread throughout the industrial world, our findings have broad implications that are directly relevant to the current level of ethnic, racial, and cultural tensions that pervade our society.

## Method

### Participants

Seventeen young healthy adults (8 females; 9 males) were enrolled in this study. Participants ranged in age between 19–31 years (M = 24.53 years, SD = 4.20). Seven participants identified as Caucasian (41.2%), eight as African American (47.1%), one participant identified as Asian (5.9%) and one as Native American (5.9%). None of the participants identified as having Hispanic/Latino ethnicity in addition to their race.

Participants were randomized to the order of experimental conditions (sleep restriction or control) on the first day of the first 25-day-hospital stay. An independent statistician prepared envelopes with randomization codes, one of which was opened by a senior staff member prior to the first hospital stay. Due to the logistics of the study, the investigators who administered the computerized IAT were not blinded to the group allocation.

### Eligibility criteria

Participants were eligible to take part if (i) they were between 18–55 years old, (ii) had a body mass index (BMI) between 18.5–30, (iii) reported their daily sleep duration to be between 7–9 hours (verified by sleep log data for two weeks prior to entry), (iv) their habitual sleep period began within one hour of 2300 h (to ensure normal entrainment), and (v) they showed blood chemistry in the normal range. Exclusion criteria included (i) active infection/disease, (ii) history of psychiatric, neurological, pain-related, immune or cardiovascular disease, (iii) significant allergy, (iv) Raynaud’s syndrome, (v) pregnancy/nursing, (vi) respiratory disturbance index of >5 events/hour on a polysomnographic sleep study, and/or leg movement with arousal >10/hour, and/or sleep efficiency <80% (i.e., findings indicative of a sleep disorder), and (vii) regular medication use other than oral contraceptives.

### Arab-Muslim Names Implicit Association Test (IAT)

The version of the IAT used here was the Arab-Muslim IAT from Harvard’s Project Implicit^®^ (https://implicit.harvard.edu/implicit/selectatest.html). The IAT is a computerized task designed to measure negative implicit biases towards Arab-Muslim names. Participants completed the task on a laptop computer on Day 21 of each of the two conditions. As part of the instructions, participants were told that they would be presented with a set of words to classify into two groups. They were also told that this task required them to respond to Arab Muslim names and names of Other People. Participants were given the opportunity to discontinue the task if it made them uncomfortable; however all participants chose to complete the task on both occasions. Participants were then told that this task measured their reaction speed and that they should work as fast as possible without making mistakes.

The IAT was composed of five blocks. The first two blocks and the forth block were practice blocks during which participants were presented with one type of category (i.e., either names or valence). For the names practice block, the categories “Other People” were presented on one corner of the screen and “Arab Muslims” were presented on the other corner of the screen. Participants were asked to categorize first names (e.g., Arab Muslim: “Yousef”; Other people: “Philippe”) into these two categories by pressing the left or right response keys. In the second practice block, participants were presented with the categories “Positive” and “Negative” and asked to categorize positive (e.g., “Wonderful) and negative words (e.g., “War”) into these two categories. After these two practice blocks, participants were then presented with the first critical trial (e.g., the “congruent” trial) where participants were presented with the categories “Other people or Positive” and “Arab Muslims or Negative” and were asked to categorize names and positive and negative words into these two categories. Participants then completed another practice run of categorizing negative and positive words. The final critical trial (e.g., the “incongruent” trial) asked participants to sort names and positive and negative words into the categories “Other people and Negative” and “Arab Muslims and Positive”. The two critical blocks included 40 trials whereby each name and word was presented once in a random order. The order in which the critical blocks were presented was counterbalanced across participants, and across time points.

Scores on the IAT were analyzed in line with Greenwald *et al*.’s^25^ improved scoring algorithm in order to obtain a “d” score. First, consistent with this approach, all trials greater than 10,000 msec were deleted. Then, all subjects for whom more than 10% of trials had a latency of less than 300 msec were also deleted. One participant responded with such extreme values in more than 20% of their responses, and was therefore excluded from the analysis. In the next step, the mean latency of correct responses for the two critical trials was calculated, and a penalty reaction time score was calculated (i.e., mean for correct responses + 600 msec) and used for all incorrect responses. Then, one “inclusive” standard deviation for all trials in both of the critical blocks (i.e., congruent and incongruent) was calculated. Finally, the mean difference score (incongruent trials – congruent trials) was divided by the inclusive standard deviation. The resulting d-score was used in the analysis. The d-score has a possible range of −2 to +2. A d-score of zero indicates no IAT effect, positive scores indicate a slight (~0.15), moderate (~0.35), or large (~0.65) IAT effect^25^. Negative scores would indicate a preference for “Arab Muslims” over “Other People”.

### Sleep restriction paradigm

Eligible participants underwent two 25-day in-hospital stays (restricted sleep condition and sleep control condition). The study paradigm is described in detail in Simpson, *et al*.^33^. In brief, participants stayed in a private or semi-private room in the Clinical Research Center (CRC) at Beth Israel Deaconess Medical Center in Boston, MA. During the rested sleep control condition, participants slept for 8 hours per night (2300 h–0700 h) every night for 3 weeks. During the sleep-restricted condition, participants were allowed to sleep for 4 hours (0300–0700 h) for 5 nights followed by 2 nights of recovery sleep of 8 hours. This pattern was repeated three times over the 25-day period. The two visits were scheduled at least 2 months apart and the order was counterbalanced across participants. Participants underwent intensive physiological recordings on seven out of the 25 days (i.e., on the first night, every fifth day of restricted/control sleep and every second night of recovery/control sleep in each of the three weeks) (not under investigation in the present study; see ref. 33). On Day 21 (i.e., the last sleep-restricted day of the last cycle or after 21 nights of 8 hours of sleep), participants completed the IAT at 1500 h.

### Ethical considerations

This study was approved by the Institutional Review Board (IRB) for the Protection of Human Subjects at Beth Israel Deaconess Medical Center. Participants were recruited using community advertisements. All methods were performed in accordance with the relevant ethical guidelines and regulations. All participants provided written informed consent before participating in the study and were compensated for their time.

### Power Analysis

A priori power analyses were conducted using G*Power^34^. The power analysis showed that, assuming a large effect size (f = 0.45) with sufficient power (β = 0.80) at α = 0.05, and assuming a correlation among repeated measures of r = 0.31, a total sample size of N = 14 would be needed to conduct a repeated measures within-factors analysis of variance to compare performance on the IAT during the sleep control and sleep restricted condition.

Predicted effect size was estimated using data from a previous study that found a large effect size for observing an IAT effect (d = 1.01)^35^ and an additional study that found that implicit biases towards Black individuals can be changed after administration of Propranolol (d = 0.80), without affecting explicit racial attitudes^36^. Correlation among repeated measures were estimated based on a previous study that showed a correlation of r = 0.31 between two measurements on the IAT that occurred two weeks apart^37^.

### Data Availability Statement

The datasets generated during and/or analysed during the current study are available from the corresponding author on reasonable request.
